# Relaxed DNA substrate specificity of transposases involved in programmed genome rearrangement

**DOI:** 10.1093/nar/gkaf577

**Published:** 2025-07-10

**Authors:** Matt W G Walker, Takahiko Akematsu, Erhan Aslan, Danylo J Villano, Harrison S Fried, Hui Lan, Samuel H Sternberg, Laura F Landweber

**Affiliations:** Department of Biological Sciences, Columbia University, New York, NY 10027, United States; Department of Biochemistry and Molecular Biophysics, Columbia University, New York, NY 10032, United States; Department of Biochemistry and Molecular Biophysics, Columbia University, New York, NY 10032, United States; Department of Biology, Faculty of Science, Kanagawa University, Yokohama, Kanagawa 221-8686, Japan; Department of Biochemistry and Molecular Biophysics, Columbia University, New York, NY 10032, United States; Department of Biochemistry and Molecular Biophysics, Columbia University, New York, NY 10032, United States; Department of Biochemistry and Molecular Biophysics, Columbia University, New York, NY 10032, United States; Department of Biology, Barnard College, Columbia University, New York, NY 10027, United States; Department of Biochemistry and Molecular Biophysics, Columbia University, New York, NY 10032, United States; Howard Hughes Medical institute, Columbia University, New York, NY 10032, United States; Department of Biological Sciences, Columbia University, New York, NY 10027, United States; Department of Biochemistry and Molecular Biophysics, Columbia University, New York, NY 10032, United States

## Abstract

During post-zygotic development, the ciliate *Oxytricha trifallax* undergoes massive programmed genome rearrangement that involves over 225 000 DNA cleavage and joining events. An *Oxytricha* family of Tc1*/mariner* transposons, known as telomere-bearing elements (TBEs), encodes a transposase that has been implicated in rearrangement, but its high copy number (>34 000 paralogs) has precluded genetic strategies to investigate its DNA recognition properties directly in *Oxytricha*. Here, we developed a heterologous strategy to assay TBE transposase expression and activity in *Escherichia coli*, revealing highly promiscuous DNA cleavage properties. Systematic ChIP-seq experiments allowed us to define the DNA binding specificities of multiple distinct transposase subfamilies, which exhibited a binding and cleavage preference for short, degenerate sequence motifs that resemble features present within the TBE transposon ends. The relaxed sequence preference is striking for autonomous transposases, which typically recognize their end sequences with strict specificity to avoid compromising host fitness. Finally, we developed a custom antibody to investigate TBE transposases in their native environment and found that they precisely localize to the developing nucleus exclusively during the rearrangement process. Collectively, this work establishes a robust heterologous workflow for the biochemical investigation of enzymes that have been repurposed for large-scale genome rearrangements.

## Graphical Abstract

**Graphical Abstract:**
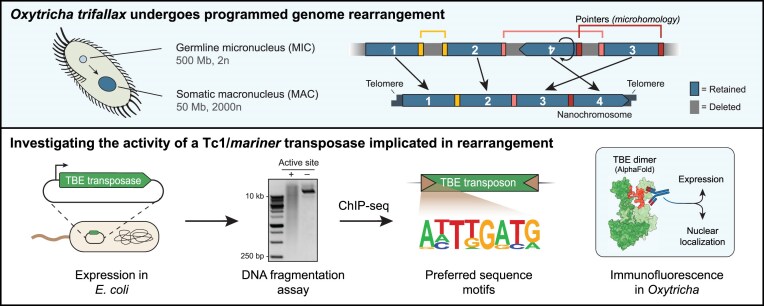

## Introduction

The genomes of many organisms undergo programmed rearrangements that require precise DNA elimination and assembly [1, 2]. Arguably, the most complex programmed genome rearrangements occur in the ciliated protozoan, *Oxytricha trifallax*. Like most ciliates, *Oxytricha* is binucleated, with a separate germline micronucleus (MIC) and somatic macronucleus (MAC) [3]. During the vegetative (asexual) life cycle, the MIC genome replicates through mitosis, while the MAC genome divides through amitosis, with chromosomes imperfectly segregated to the daughter nuclei. During sexual reproduction, conjugating cells exchange haploid micronuclei that undergo syngamy, the parental MAC degrades, and one copy of the zygotic germline MIC develops into a new MAC, initiating an intricate cascade of recombination events requiring over 225 000 DNA cleavage and joining events [3–5]. This rearrangement process also results in the full elimination of >90% of germline DNA.

**Figure 1.**
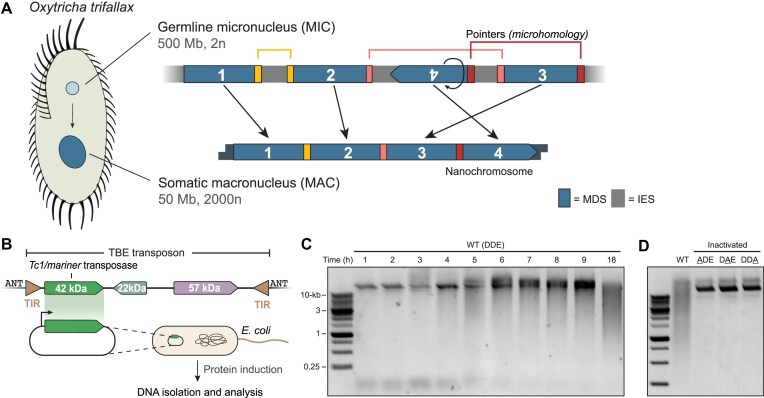
The TBE transposase induces DNA fragmentation in *Escherichia coli*. (**A**) Genome rearrangement in *Oxytricha trifallax*. *Oxytricha* cells contain a germline micronucleus (MIC), which is diploid (2*n*) and encodes ∼500 Mb of genome content. The somatic macronucleus (MAC) is polyploid (2000*n*) and encodes ∼50 Mb of genome content. Numbered, blue rectangles illustrate MDSs, which are separated by IESs, gray, in the MIC. During rearrangement, IESs are eliminated and MDSs rearrange at short homologous sequences (pointers, color-matched small vertical bars) to form functional gene-length nanochromosomes. (**B**) Schematic of TBE transposon genetic architecture (top), and heterologous TBE transposase expression approach in *E. coli*. Terminal inverted repeats (TIRs) are indicated by inverted triangles, which are flanked by the ANT target-site duplication. DNA fragmentation analysis upon TBE transposase induction in *E. coli*, using either the WT transposase with intact DDE motif (**C**) or catalytically inactivated transposase mutants bearing either an ADE mutation or DAE or DDA (**D**). In panel (C), samples represent a time course of WT induction, as labeled. In panel (**D**), DNA was isolated after 18 h of induction. Five hundred nanograms of DNA was separated by electrophoresis on a 1% agarose gel and visualized using SYBR Safe.

The MIC is present in multiple copies per cell and is largely transcriptionally silent, in part because a large portion of its precursor gene segments are in the wrong order relative to the transcribed version in the MAC; individual gene segments are also interrupted by internally eliminated sequences (IESs) that must be removed in order for retained macronuclear-destined sequences (MDSs) to arrange to produce functional MAC genes (Fig. 1A). In the MIC, MDSs are flanked by short direct repeats, called pointers, only one of which remains after rearrangement. MDS joining produces >16 000 new “nanochromosomes” that each acquire two telomeres and are amplified to high copy number (average ploidy ∼1900), exhibit an average length of 3.2 kb, and usually encode only one gene [3, 5–7]. Previous studies identified key molecular components that are involved in the rearrangement process: 27-nt Piwi-interacting small RNAs (piRNAs) mark MDSs for retention [8], long noncoding RNAs transcribed from parental MAC chromosomes program the MDS assembly process [9], and a large family of germline-encoded transposases are essential for DNA processing [10].

TBE, or telomere-bearing element, transposons are abundant in the MIC, present at >34 000 copies that comprise almost 15% of the germline genome, but they are completely eliminated during development. TBEs encode three distinct ORFs, one of which is a 42-kDa transposase (Fig. 1B). Phylogenetic analyses indicated that the TBE transposase belongs to the Tc1/*mariner* family [11], which uses a DDE catalytic triad to perform the DNA cleavage and strand joining chemistry required for transposition [12]. Phylogenetic analyses also revealed that there are three superfamilies for the TBEs—TBE1, TBE2, and TBE3—with TBE2 further subdivided into TBE2.1 and TBE2.2 [6]. The TBE moniker originates from the TIR sequences flanking the elements, which encode telomere-like repeats (G_4_T_4_)_4_. Tc1/*mariner* family elements typically contain inverted repeats at their end sequences, which delineate the boundaries of the mobile element and recruit the transposition machinery with strict specificity during mobilization. In *Oxytricha*, TBEs are detected as extrachromosomal circular DNA during genome rearrangement, indicating that they are excised as circular products [13, 14], although direct mobilization activity has not been experimentally observed. While TBE transposons likely spread and proliferate via transposition, extant TBE transposases may instead play a predominant role in DNA excision, akin to the role of RAG1 recombinase in V(D)J recombination, more often removing rather than mobilizing genomic elements. Unlike Tc1/*mariner* elements, which recognize a TA target site and are flanked by two copies of that motif after integration, TBEs are flanked by an ANT motif, which is strikingly consistent with the most common motif of 3-bp pointer sequences [4]. This overlap suggests that the motif is either a requirement for transposase-mediated DNA elimination or a remnant of transposon integration events that led to the emergence of IESs [15].

Previous work on TBEs indicated their involvement in programmed DNA elimination. Specifically, RNAi knockdown of transposase gene families led to stalled rearrangement of MAC genomic loci, arrested development, and the accumulation of high molecular weight DNA along with incompletely or improperly rearranged genes [10]. However, failed rearrangement only occurred when RNAi was directed against all 3 superfamilies of TBEs, limiting the utility of this approach to investigate the activity of specific TBE-encoded transposases. Furthermore, while both reverse and forward genetic tools in *Oxytricha* can target the MAC [8, 16], techniques for genetic manipulation of the germline MIC are severely compromised by its low copy number and small size compared to the MAC, and this severely limits our ability to determine how and where TBE transposases cleave DNA to effect precise DNA elimination during development.

To circumvent these limitations, we developed a heterologous expression approach in *E. coli*, which allowed us to gain insights into DNA binding and cleavage activity of the TBE transposase. We uncovered DNA binding preferences within the *E. coli* genome and its own TBE transposon ends, and find that this binding activity is consistent with DNA cleavage, which we detected using deep sequencing to profile fragmented DNA. Our work presents the first direct evidence of TBE transposase binding and cleavage activity, and establishes a robust workflow for studying a previously intractable enzyme implicated in genome rearrangement.

## Materials and methods

### Plasmid and *E. coli* strain construction

Plasmids and oligonucleotides used in this study are listed in Supplementary Tables S1 and S2, respectively. *Escherichia coli* codon-optimized open reading frames for TBE transposon-encoded proteins were synthesized and cloned into expression vectors under the control of an IPTG-inducible T7 promoter on medium-copy pET vectors with carbenicillin resistance. Epitope-tagged protein expression vectors were constructed by appending a C-terminal FZZ tag (3× FLAG-tag, TEV protease cleavage size, and ZZ domain of protein A, described in Kataoka *et al.*, 2010 [17]). Catalytically inactivated derivatives of the TBE transposase proteins were constructed by “around-the-horn” polymerase chain reaction (PCR), to mutate each residue of the catalytic triad (DDE) to alanine (ADE, DAE, and DDA). All plasmids were cloned into NEB Turbo cells (NEB), purified (QIAprep Spin Miniprep Kit), and verified by Sanger sequencing (GENEWIZ).

Four custom *E. coli* BL21 (DE3) strains were generated encoding a genomically integrated TBE transposon belonging to each of the TBE families (TBE1 from contig ctg7180000067530, TBE2.1 from contig 7180000067905, TBE2.2 from contig ctg7180000069065, and TBE3 from ctg7180000089059). These strains were generated by Himar1C9-mediated transposition. We cloned representative TBE transposons from the four TBE families onto a temperature-sensitive pSC101 plasmid, downstream of a kanamycin resistance cassette, which was flanked by *mariner*-family transposon end sequences. The plasmid also encoded a transposase expression cassette, which produced the Himar1C9 protein required for transposition of the *mariner* element. The temperature-sensitive plasmid was cured before selecting for antibiotic resistance, which could only be conferred after genomic integration of the TBE element. Specifically, after transformation by heat shock, cells were recovered at the restrictive temperature and plated on LB agar plates supplemented with kanamycin (50 μg/ml), and colonies that grew were confirmed by PCR to have lost the temperature-sensitive TBE plasmid. The genomic integration was later confirmed for each of the four strains by Illumina deep sequencing (Smear-Seq and ChIP-seq), which detected a single copy of the transposon in the genome and revealed the integration site, allowing us to generate complete *E. coli* reference genomes for each strain that were used for read mapping and analysis.

### DNA fragmentation assay and time course experiments

The DNA fragmentation analysis relied on inducing transposase expression in liquid media, performing a miniprep to isolate DNA, and separating DNA by electrophoresis on a 1% agarose gel. First, chemically competent *E. coli* BL21 (DE3) cells were transformed and single colonies were grown overnight at 37°C in LB medium with 100 μg/ml carbenicillin. The next day, cultures were diluted 1:100 in fresh LB medium with 100 μg/ml carbenicillin and grown at 37°C until mid-logarithmic phase (OD_600_= 0.4). Cultures were then induced with 0.1 mM IPTG and transferred to 16°C. For the time course experiments, multiple 2 ml cultures were set up in replicate. After the defined induction time, cell density was measured (OD_600_), DNA was isolated (QIAGEN Plasmid Mini Kit), and DNA concentration was measured (DeNovix Spectrophotometer). Five hundred nanograms of DNA was loaded onto each lane of a 1% agarose gel stained with SYBR Safe (Thermo Fisher Scientific), subjected to electrophoresis (130 V for 20 min), and imaged (Bio-Rad GelDoc XR Imaging System).

### Structural predictions and sequence alignments

Structural models of the TBE transposase dimer were predicted using AlphaFold version 2.3.1 (commit 18e12d6) [18], using the multimer model preset (--model_preset=multimer) [19], and displayed in ChimeraX (v1.6). Structural models of the TBE transposase dimer interacting with 40 bp of transposon end DNA were predicted using RoseTTAFoldNA [20]. Multiple sequence alignments were generated by Clustal Omega run on EMBL’s Job Dispatcher [21], and visualized in Jalview version 2.11.3.2 [22].

### NGS approach for sequencing fragmented DNA (“Smear-seq”)

**Figure 2.**
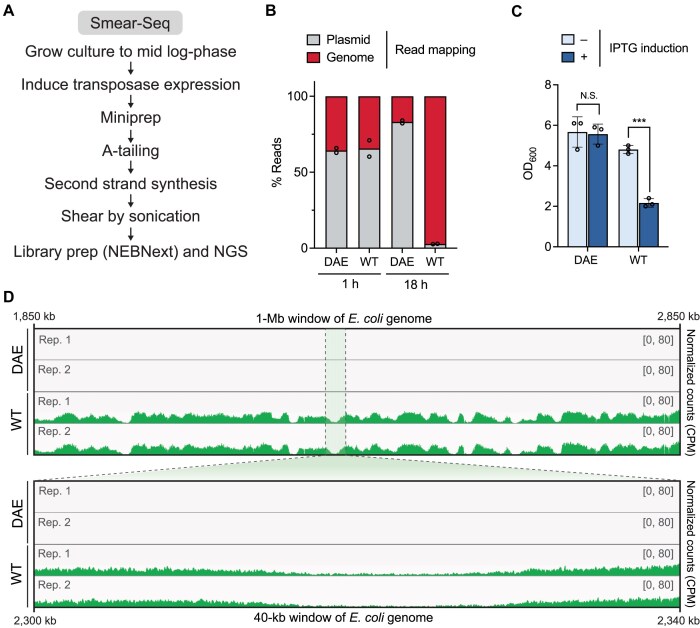
The TBE transposase cleaves genomic DNA and induces cell death. (**A**) “Smear-seq” workflow for sequencing fragmented DNA in *E. coli*. (**B**) Percentage of reads from each sample mapping to the transposase expression plasmid or to the *E. coli* genome. Each point represents one independent biological replicate. (**C**) Optical cell density (OD_600_) of the WT or inactivated (DAE) transposase after 18 h with and without IPTG induction. Data are shown as mean ± SD for *n* = 3 biological replicates; ***P < .001 (unpaired *t*-test); N.S., not significant. (**D**) Read coverage across the *E. coli* genome in a 1-Mb window (top) or 40-kb window (bottom). The *y*-axis represents read counts per million (CPM), with the axis limit set to 80 for each sample.

Our approach to sequencing fragmented DNA relied on DNA isolation, dA-tailing, second-strand synthesis, sonication, library prep, and next-generation sequencing (Fig. 2A). First, the wild-type or catalytically inactivated transposase was expressed as in the DNA fragmentation assay, wherein we diluted overnight cultures of *E. coli* BL21 (DE3) cells expressing the transposase 1:100 in LB with 100 μg/ml carbenicillin, induced mid-logarithmic phase cultures with 0.1 mM IPTG, and grew cultures for 18 h at 16°C. DNA was isolated (QIAGEN Plasmid Mini Kit), and A-tailing was performed using an approximate molar ratio of 1:1000 of DNA:dATPs (NEB Terminal Transferase). After incubating at 37°C for 30 min and heat-inactivating at 70°C, DNA was cleaned and eluted in 40 μl Milli-Q H_2_O (QIAquick PCR Purification Kit). Concentration was measured by fluorometry (DeNovix dsDNA High Sensitivity Assay) before second-strand synthesis using oligo (dT)_18_ primers and PrimeSTAR Max DNA Polymerase (Takara). DNA was cleaned and eluted in 40 μl Milli-Q H_2_O and the concentration was measured by fluorometry before normalized samples were loaded into a microTUBE AFA Fiber Crimp-Cap and TE buffer was added to a total volume of 130 μl. Samples were then sheared by sonication on an M220 Focused-ultrasonicator (Covaris) using the following SonoLab 7.2 settings: peak power, 50.0; duty factor, 20; cycles/burst, 200; and treatment time, 150 s. After sonication, DNA was prepared for NGS using the NEBNext Ultra II DNA Library Prep for Illumina (NEB). After adaptor ligation, DNA was purified without size selection using AMPure XP beads (Beckman Coulter) and appended sequencing indexes with 10 cycles of PCR and NEBNext Q5 Master Mix. Two-sided size selection was used to clean and select ∼450-bp fragments. To first remove small fragments, 0.55× AMPure XP bead solution was added and samples were allowed to separate on a magnetic rack before the supernatant containing small DNA fragments was removed. Then, to remove large fragments, 0.35× AMPure XP bead solution was added to each sample, separated on a magnetic rack, and the supernatant discarded. Beads were then washed twice in 80% (v/v) EtOH and eluted in 12 μl of TE buffer. Concentration was determined by fluorometry (DeNovix dsDNA High Sensitivity Kit) and samples were sequenced using a NextSeq High Output Kit with 150 cycles (Illumina).

After sequencing, paired-end reads were filtered and trimmed using fastp [23] and mapped to the *E. coli* BL21 (DE3) reference genome and to the TBE expression plasmid using bowtie2 [24]. Then, we used Samtools to sort, index, and filter multi-mapping reads [25]. Finally, coverage was normalized to CPM using the deepTools2 command bamCoverage [26].

### *Oxytricha* culture conditions and mating

*Oxytricha* strains were cultured and mated as in [8]. Two wild-type *Oxytricha* strains with different mating types, JRB310 and JRB510, were cultured in Pringsheim media [0.11 mM Na_2_HPO_4_, 0.08 mM MgSO_4_, 0.85 mM, Ca(NO_3_)_2_, 0.35 mM KCl, pH 7.0] supplemented with *Chlamydomonas reinhardtii* and *Klebsiella* as the food source. To induce mating, cells were allowed to deplete *Chlamydomonas* overnight, filtered through cheesecloth to remove any remaining debris, and equal amounts of cells were mixed at a density of 5000 cells/ml with a final volume of 300 ml. Pairs typically form 3–4 h after mixing, and maximum pairing efficiency (∼70–80%) is generally achieved 12 h post-mixing.

### Production of anti-TBE transposase antibodies

The peptide KRQHLNSKPKRPLK was synthesized and used for immunization in rabbits to yield anti-TBE transposase antibodies (GenScript). Rabbits were immunized three times at 2-week intervals. 4.28 mg antibody was purified and suspended in 7.5 ml phosphate buffered saline (PBS) with 0.02% ProClin 300 preservative. The antibody was validated by western hybridization in *E. coli* cells expressing the TBE transposase, as described below.

### Western blotting of heterologous TBE protein expression in *E. coli*

Protein expression in *E. coli* was confirmed by western analysis, which was performed essentially as previously described [27] with minor modifications. *Escherichia coli* overnight cultures expressing FZZ- or MBP-tagged proteins were diluted 1:100 in LB supplemented with 100 μg/ml carbenicillin and grown at 37°C to OD_600_= 0.4. Protein expression was induced with 0.1 mM IPTG, and cultures were transferred to 16°C and grown for 18 h. The equivalent of 1 ml of OD_600_= 1.5 culture was pelleted by centrifugation (12 000 × g for 5 min at 4°C), and the pellet was suspended in 150 μl lysis buffer (20 mM Tris–Cl, pH 7.5, 150 mM NaCl, 0.5 mg/ml lysozyme). Samples were incubated at 25°C for 10 min before adding 10 mM DTT, 1% *N*-lauroyl sarcosine, and 2× SDS loading dye [100 mM Tris–Cl, pH 6.8, 4% (w/v) SDS, 30% (v/v) glycerol, and 0.07% (w/v) bromophenol blue]. Samples were incubated at 95°C for 10 min, and 5 μl of each sample underwent separation on a 4%–12% gradient sodium dodecyl sulfate–polyacrylamide gel electrophoresis (SDS–PAGE) gel (Bio-Rad, Mini-PROTEIN TGX) at 100 V for 90 min. Proteins were transferred to a PVDF (polyvinylidene difluoride) membrane (Invitrogen iBlot 2 Transfer Stack). Subsequent washing, blocking, and antibody incubation were performed by gentle nutation at room temperature (RT). Membranes were washed with 1× PBS + 0.1% Tween 20, then incubated for 1 h in blocking buffer [1× PBS, 0.1% Tween 20, 5% bovine serum albumin (BSA)], washed in 1× PBS + 0.1% Tween 20, and then incubated in primary antibody diluted in blocking buffer for 1 h at RT. For the FZZ-tagged samples, 1:40 000 monoclonal anti-FLAG M2 antibody produced in mouse (Sigma–Aldrich, F1804) was used. For the MBP-tagged sample, 1:10 000 of anti-TBE transposase antibody (GenScript) was used. For the loading control, 1:5000 anti-GAPDH monoclonal antibody produced in mouse (Invitrogen, MA5-15738) was used. Membranes were washed three times in 1× PBS + 0.1% Tween 20, and primary antibodies were detected with either goat anti-mouse IgG1 heavy chain HRP (Abcam, ab97240) or goat anti-rabbit IgG (H + L)-HRP (Bio-Rad, 1706515). Finally, we performed enhanced chemiluminescence (Thermo Fisher SuperSignal West Dura Extended Duration Substrate) and imaged blots on an Amersham Imager 600 (GE).

### Western blotting of native TBE protein expression in *Oxytricha*

Approximately 300 000 mating cells from different time points were collected by centrifugation at 130 × *g* for 2 min using a swinging bucket centrifuge (Sorvall RC6, Thermo Scientific). The supernatant was discarded, and the cell pellet was suspended in the residual medium. Next, 2× SDS loading buffer was directly added to the samples, which were then heated at 95°C for 10 min. The samples were run on TGX Stain-Free Precast Gels (Bio-Rad) and transferred to a 0.2-μm Immun-Blot PVDF membrane (Bio-Rad, 1620177) under semi-dry conditions using Trans-Blot SD blotter. The membrane was blocked with 5% nonfat dry milk (biokemix, M0841) in 1× TBS-T for 1 h at RT and then incubated overnight at 4°C on a rotator with a custom-made rabbit anti-TBE transposase antibody (1:1000 dilution in blocker). The following day, the membrane was washed five times with 1× TBS-T (5 min each) and incubated with 1:10 000 goat anti-rabbit IgG (H + L)-HRP (Bio-Rad, 1706515) for 1 h at RT. After final washes (five times with 1× TBS-T, 5 min each), the chemiluminescence signal was detected using Clarity Western ECL reagent (Bio-Rad), and the membrane was imaged with a ChemiDoc imager (Bio-Rad).

### Chromatin immunoprecipitation followed by next-generation sequencing in *E. coli*

Chromatin immunoprecipitation followed by next-generation sequencing (ChIP-seq) in *E. coli* was performed essentially as described [28–30], with some modifications. Cultures were prepared and protein expression was induced as described in the western blot method above. After growing induced cultures at 16°C for 18 h, formaldehyde was added to a final concentration of 1% to each 40 ml of culture and nutated for 20 min at RT. Formaldehyde was quenched by adding 4.6 ml of 2.5 M glycine, nutating for 10 min. Samples were then centrifuged at 4000 × g at 4°C for 8 min. The cell pellet was washed twice in cold 1× TBS, and then the equivalent of 40 ml of OD_600_= 0.6 was transferred to a tube to normalize cell density across samples and pelleted again by centrifugation. The pellet was transferred to a 1.5-ml Eppendorf tube and centrifuged at 10 000 × g at 4°C for 5 min. The supernatant was discarded and cell pellets were flash-frozen in liquid nitrogen.

Cell pellets were resuspended in lysis buffer [50 mM HEPES–KOH, pH 7.5, 0.1% (w/v) sodium deoxycholate, 0.1% (w/v) SDS, 1 mM EDTA, 1% (v/v) Triton X-100, 150 mM NaCl, 1× cOmplete Roche Protease Inhibitor] and transferred to a 1-ml milliTUBE AFA Fiber (Covaris). Samples were sonicated on an M220 Focused-ultrasonicator (Covaris) with the following SonoLab 7.2 settings: minimum temperature, 4°C; set point, 6°C; maximum temperature, 8°C; peak power, 75.0; duty factor, 10; cycles/burst, 200; and sonication time, 17.5 min. After sonication, 10 μl of sheared cleared lysate was transferred to a separate tube as the pre-IP control (“input”). The remainder was used for immunoprecipitation.

For immunoprecipitation, 25 μl Dynabeads Protein G (Thermo Fisher Scientific) for each sample was washed four times with 1× PBS + 0.5% BSA and mixed with 4 μl monoclonal anti-FLAG M2 antibody produced in mouse (Sigma–Aldrich, F1804), which was conjugated at 4°C for 6 h. After conjugation, beads were washed four times with 1× PBS + 0.5% BSA, resuspended in 30 μl lysis buffer, then mixed with sonicated samples, and rotated overnight at 4°C.

The following day, beads were washed three times with lysis buffer lacking protease inhibitor, then washed once with lysis buffer lacking protease inhibitor, and supplemented with 500 mM NaCl, once with ChIP wash buffer [10 mM Tris–HCl, pH 8.0, 250 mM LiCl, 0.5% (w/v) sodium deoxycholate, 0.5% (v/v) Nonidet P-40, 1 mM EDTA] and finally twice with TE buffer (10 mM Tris–HCl, pH 8.0, 1 mM EDTA). After the final wash, beads were suspended in 200 μl elution buffer [1% (w/v) SDS, 0.1 M NaHCO_3_] and incubated at 65°C for 1 h 15 min to release protein–DNA complexes from the beads, vortexing every 15 min to resuspend the beads. While incubating the immunoprecipitated samples, 10 μl of the frozen pre-IP samples were withdrawn and 190 μl elution buffer was added. After the 65°C incubation was complete, 10 μl of 5 M NaCl was added to each pre-IP and IP sample, which were incubated at 65°C overnight to reverse crosslinks.

After overnight incubation, RNA was digested by adding 1 μl RNase A (Thermo Fisher Scientific) and incubating at 37°C for 1 h. Protein was digested by adding 2.8 μl of 20 mg/ml proteinase K (Thermo Fisher Scientific) and incubating at 55°C for 1 h. DNA was purified (QIAquick PCR Purification Kit) and eluted in 40 μl TE buffer before the concentration was measured by fluorometry (DeNovix dsDNA Ultra High Sensitivity Kit). Samples were normalized to the lowest concentration, and DNA was prepared for next-generation sequencing using the NEBNext Ultra II DNA Library Prep Kit for Illumina (NEB). ChIP-seq samples were sequenced on a paired-end run using NextSeq Mid or High Output Kits with 150 cycles (Illumina).

Paired-end reads were filtered, trimmed, and mapped as described in the Smear-seq workflow above. After read mapping, peaks were called using MACS2 [31], filtered to exclude peaks with a score <20, and *de novo* motif prediction was performed using Homer using the command “findMotifsGenome.pl” with default settings and a window size of 100 [32].

### ChIP-seq in *Oxytricha*

*Oxytricha* cell lysates were prepared for ChIP-seq at 0, 36, and 48 h after mixing of cells of compatible mating type. Two million cells were fixed in 1% formaldehyde (Sigma) for 2 min, and crosslinking was quenched by adding glycine to a final concentration of 125 mM, followed by incubation for 5 min at RT on a rotator. Fixed cells were then lysed in 1 ml of ChIP lysis buffer (50 mM HEPES–KOH, pH 7.5, 140 mM NaCl, 1 mM EDTA, 1% Triton X-100, 1% SDS, 0.1% sodium deoxycholate, 1× Protease Inhibitor Cocktail, Sigma) for 15 min on ice. Chromatin was sheared using a Q800R3 Sonicator with 25 cycles of 30 seconds on/off with 40% amplitude, spun down, and the cleared lysate was flash frozen at −80°C.

Each 1 ml sample was diluted 10× in dilution buffer (50 mM Tris–HCl, pH 8.0, 150 mM NaCl, 1 mM EDTA, pH 8.0, 1% Triton, 1× cOmplete Roche Protease Inhibitor) and pre-cleared in Dynabeads Protein G. To bind the anti-TBE transposase antibody to the beads, 50 μl Dynabeads Protein G for each sample was washed two times with 1× PBST and mixed with 17.5 μl of custom anti-TBE transposase antibody produced in rabbits, which was conjugated at 4°C for 6 h. After conjugation, beads were washed once with 1× PBST, resuspended in 50 μl dilution buffer, then mixed with the sonicated pre-cleared samples, and rotated overnight at 4°C. The following day, the remaining ChIP-seq steps (protein/RNA digestion, DNA cleanup, and NGS library preparation) were performed as described above, in the “Materials and methods” section for *E. coli* ChIP-seq.

### Indirect immunofluorescence

Immunofluorescent staining experiments were performed as previously described [8] with minor modifications. Briefly, cells were fixed with 4% paraformaldehyde (Electron Microscopy Sciences) for 10 min at RT on an end-to-end rotator, followed by two washes with 1× PBS. Fixed cells were placed on a poly-l-lysine-coated (0.1 mg/ml, Gibco) hydrophobic printed 12-well slide (Epredia, ER202W) and incubated overnight at 4°C. The cells were then permeabilized with permeabilization solution (0.5% Triton X-100 in 1× PBS) for 30 min at RT, followed by a 5-min incubation in 0.1 M HCl. After a brief wash with 1× PBS, Image-iT™ FX Signal Enhancer (Invitrogen) was applied, and the cells were incubated for 30 min at RT. Cells were subsequently blocked with blocking buffer (0.2% Triton X-100, 0.5% IgG, and protease-free BSA in 1× PBS) for 1 h at RT. Rabbit anti-TBE transposase antibody, diluted 1:100 in blocking buffer, was applied to the cells, and the slide was incubated overnight at 4°C. The next day, the cells were washed three times with washing solution (0.2% Triton X-100 in 1× PBS) for 10 min each at RT. Alexa Fluor™ 488-conjugated goat anti-rabbit secondary antibody (1:1000 in blocking solution; Invitrogen, A11034) was then applied, and the slide was incubated at 37°C for 1 h in the dark. After a final series of three washes with washing solution (10 min each at RT), DAPI-containing VECTASHIELD^®^ Antifade Mounting Medium (Vector Laboratories, H-1000–10) was added to the samples, and the slide was covered with a long cover glass (Fisherbrand, 12545J). Fluorescent images were captured using a Zeiss Axiovert 200 inverted microscope equipped with an Axiocam 506 monochrome camera. Images were pseudo-colored using ImageJ when merging two channels.

## Results

### Expression of the TBE transposase ORF induces widespread DNA fragmentation in *E. coli*

To study the function of a high copy number transposase implicated in genome rearrangements in *Oxytricha*, we set out to heterologously express candidate TBE transposases in *E. coli*, focusing our initial efforts on a representative transposon encoded in the *Oxytricha* MIC genome and referred to as TBE2.1 (7905), a member of the TBE2.1 family encoded on contig 7180000067905 [4]. When we expressed the protein in *E. coli* BL21 (DE3) and isolated and analyzed the cellular DNA fraction (Fig. 1B), we observed a striking DNA fragmentation phenotype, characterized by a significant and reproducible DNA smear upon electrophoretic separation (Fig. 1C). Apparent DNA degradation was visible as early as 3 h after induction, and by 18 h, the DNA products ranged broadly in length. To determine whether this phenotype was merely a cytotoxic by-product of heterologous protein over expression, we cloned inactivated variants of the transposase, mutated in the predicted active site from DDE to DAE. This and other single amino acid alanine substitutions in the DDE motif abolished the DNA smearing phenotype (Fig. 1D), as did an empty vector control (Supplementary Fig. S1A), providing the first direct evidence of TBE transposase-mediated DNA cleavage activity. Of note, these experiments in *E. coli* lacked any presence of *bona fide* TBE transposon DNA substrates.

We next set out to determine the composition of DNA products that appeared after transposase induction, via a high-throughput sequencing approach here dubbed “Smear-seq” (Fig. 2A and “Materials and methods” section). To prepare samples, we induced the WT or catalytically inactivated transposase for either 1 or 18 h, extracted DNA by miniprep, and then performed 3′ polyadenylation and second-strand synthesis, followed by ligation to sequencing adaptors, and sequencing on an Illumina platform. After mapping sequencing reads to the TBE expression vector and the *E. coli* BL21 (DE3) genome, we quantified the relative composition of plasmid or genomic DNA in each sample and found that after 18 h of induction, the WT transposase samples primarily comprised genomic DNA (>95% of reads), whereas the 3 h time point and the catalytically inactivated transposase samples primarily comprised plasmid DNA (Fig. 2B). These data suggest that presence of the TBE transposase active site permits extensive genomic DNA cleavage by the transposase in *E. coli*.

To test whether genomic DNA fragmentation was accompanied by cell death, we measured cell density by spectrophotometry and observed a dramatic loss of cell density after induction of the WT transposase, but not after induction of catalytically inactivate transposase (Fig. 2C), consistent with the genomic DNA cleavage activity of the wild-type TBE enzyme.

Our deep-sequencing approach also allowed us to search for preferred DNA cleavage sites, by examining the distribution of reads across the *E. coli* reference genome. We observed reproducible features in the WT 18 h sample on megabase-scale genomic regions within the *E. coli* genome, with consistent regions of enrichment and depletion and coverage profiles that were nearly indistinguishable across two independent biological replicates (Fig. 2D). These data suggest that the TBE transposase may be preferentially cleaving consistent regions of the genome.

### ChIP-seq reveals degenerate DNA sequence motifs bound by TBE transposases

**Figure 3.**
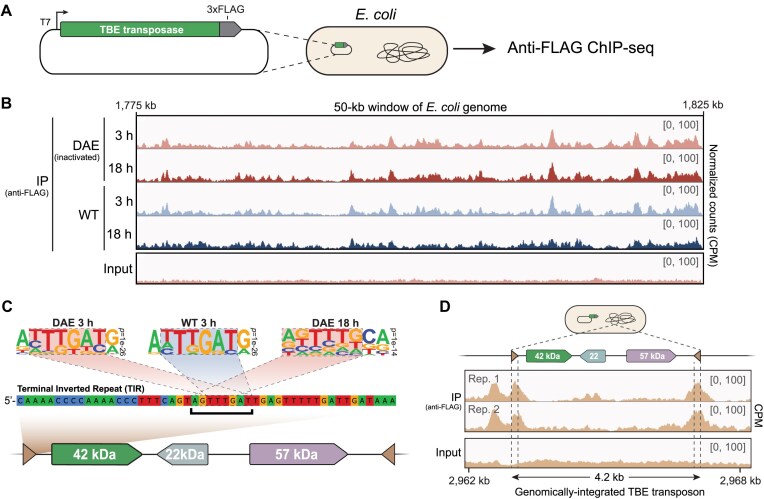
ChIP-seq reveals DNA sequence preferences for degenerate motifs partially encoded within the TBE transposon ends. (**A**) ChIP-seq approach for determining DNA binding preferences of the TBE transposase in *E. coli*. (**B**) Read coverage across a representative 50-kb window of the *E. coli* genome, for the WT and catalytically inactivated transposase (DAE). The *y*-axis represents CPM and the axis limit is set to 100. (**C**) Sequence logos represent the top statistically significant motif from *de novo* motif prediction (Homer [32]). *p*-values indicate statistical significance; motifs are considered significant if their *p*-values are <1e−10. Motif prediction for the WT 18 h sample did not detect any statistically significant motifs (N.S.). Dashed boxes indicate regions that map to the TBE transposon ends, as illustrated by the first 50 nt of the TBE2.1 TIR. (**D**) ChIP-seq read coverage using a catalytically inactive TBE2.1 transposase expressed from a plasmid for 18 h in a strain harboring a genomically integrated TBE2.1 transposon. The transposase corresponds to the version encoded within the transposon but is codon-optimized for expression in *E. coli*. Vertical dashed lines on the coverage plots indicate the TIR boundaries.

To more directly and globally profile specific DNA sites in the *E. coli* genome recognized by the TBE transposase, we applied ChIP-seq (chromatin immunoprecipitation followed by DNA sequencing) using the WT or catalytically inactivated (DAE) transposase fused to a C-terminal epitope tag (3×FLAG) (Fig. 3A), reasoning that the DAE variant would prevent DNA degradation and thus reveal the underlying substrate specificity. Examining the ChIP-seq read coverage across the *E. coli* genome revealed narrow regions of enrichment and depletion that were highly consistent across biological replicates (Fig. 3B), in stark contrast to the more diffuse Smear-seq signal (Fig. 2D). We induced each transposase variant for 3 h and 18 h and observed strong agreement between the ChIP-seq profiles for all samples excluding WT at 18 h, which had more diminished peaks, we conjecture due to accumulated DNA cleavage activity reducing the signal.

To test whether conserved DNA sequence features were present within the genomic regions of enrichment, we performed peak calling and *de novo* motif prediction. This analysis revealed a short sequence motif (5′-ANTTTGA) as the top motif detected in all samples except for WT 18 h, where no statistically significant motifs were found (Fig. 3C). This motif was called as not only the top motif in each sample, but also the only motif that met the significance threshold in each of the three samples (Supplementary Fig. S1B). The absence of statistically significant motifs in the WT 18 h sample is likely also due to transposase cleavage activity leading to cleavage and product release, thus decreasing enrichment at preferred binding sites. Intriguingly, the short sequence motif is also found within the TBE transposon TIRs (Fig. 3C), which transposases typically recognize to mobilize an element.

We hypothesized that this motif would resemble sequence features found within the TBE transposon TIR ends, and that structural modeling might identify likely points of interaction between the transposase and TIRs. To test this, we used AlphaFold Multimer [18] to predict the structure of the TBE transposase dimer. Structural alignment with the solved dimeric structure of Tc1/*mariner* transposase Mos1 revealed a similar overall fold, with a root-mean-square deviation of 7.991 Å based on Cα atom superposition (Supplementary Fig. S2A). Model quality metrics for the TBE transposase dimer prediction, including per-residue confidence scores and predicted alignment error plots, are provided in Supplementary Fig. S2B and C. Mos1, first isolated from *Drosophila mauritiana* [33, 34], encodes an N-terminal DNA binding domain (residues 1–112) and C-terminal DD[D/E]-family catalytic domain (residues 126–345). Each N-terminal DNA binding domain encodes two α-helical motifs (HTH1 and HTH2) that contact the *mariner* transposon ends at positions 21–26 and 8–13, respectively [35, 36]. Intriguingly, when we predicted the structure of the TBE transposase with 40 bp of TIR DNA (RoseTTAFoldNA [20]), its two HTH motifs were positioned near the TTT trinucleotide motifs at the transposon ends, with the DNA binding domains contacting TIR DNA at positions 24–27 and 16–18, respectively (Supplementary Fig. S2B). The TTT trinucleotide motif was also detected in the *E. coli* ChIP-seq peaks (Fig. 3C), lending additional support to the interpretation that the transposase exhibits DNA sequence preferences for this motif.

Next, we set out to test whether we could detect transposase enrichment within the native TBE transposon end sequences. We generated a genomically modified *E. coli* strain that harbored a complete TBE transposon element in its circular chromosome and performed ChIP-seq in this new genetic background (Fig. 3D). We observed strong enrichment within the transposon TIRs, offering the first evidence that the TBE transposase recognizes its transposon ends and suggesting that the TBE2.1 transposase may be actively targeting TBE elements during *Oxytricha* genome rearrangement.

To test whether the transposase could also recognize *Oxytricha* genomic elements that are eliminated during programmed DNA rearrangement, we cloned an ∼4.5-kb region of micronuclear DNA covering the TEBPβ gene locus, which has the most thoroughly studied rearrangement products [9, 10]. To avoid reintegrating the *Oxytricha* genomic element into the *E. coli* genome, we first tested whether we could recapitulate ChIP-seq results with a plasmid-encoded TBE transposon element, and indeed observed consistent enrichment profiles between the genomic- and plasmid-encoded elements (Supplementary Fig. S3A). When we examined the enrichment profile across the native TEBPβ locus, introduced on a bacterial plasmid, we observed a variable pattern of enrichment clustered near some eliminated regions, particularly those that are more densely packed (Supplementary Fig. S3B), with read coverage highest in a region containing a high density of rearrangement junctions and IESs. This suggests that precise targeting of the transposase to eliminated sequences in *Oxytricha* may rely on chromatin features or protein cofactors that are absent in *E. coli*, such as heterochromatin-associated marks or other recruitment mechanisms.

### TBE accessory proteins do not alter TBE DNA binding profiles in *E. coli*

The TBE transposon encodes two proteins in addition to the putative transposase (Fig. 1B), which are under purifying selection and are proposed to function as accessory factors during transposition [6]. This feature is surprising for Tc1/*mariner* elements, which typically encode only their transposase and generally lack accessory proteins [37]. One of the two *Oxytricha* proteins is estimated to be 57 kDa and comprises predicted zinc-finger and kinase domains (Supplementary Fig. S4A), while the other is estimated to be 22-kDa protein with no predicted domains [6] (Supplementary Fig. S4B). To assay in *E. coli* whether the expression of these accessory proteins could affect DNA cleavage or binding of the TBE transposase, we prepared epitope-tagged expression vectors for each component and co-expressed them with untagged dual-expression vectors for the other components (Supplementary Fig. S4C). We first confirmed protein expression of each element by western blot and observed bands at the expected size for each protein, as well as a second, smaller band for the 57-kDa sample that might represent a protein degradation product (Supplementary Fig. S4D). ChIP-seq against tagged versions of either accessory protein or the TBE transposase, in the presence or absence of the two other untagged proteins, did not display differences in the DNA binding profiles for any combination relative to the pre-IP input profile, suggesting that the accessory proteins might not bind DNA with specificity (Supplementary Fig. S4E) or that the *E. coli* expressed versions might not capture the *Oxytricha* environment or modifications necessary for function. It is also possible that the accessory proteins participate in later steps of transposition or repair during genome rearrangement.

### Transposase homologs exhibit similar DNA sequence preferences

**Figure 4.**
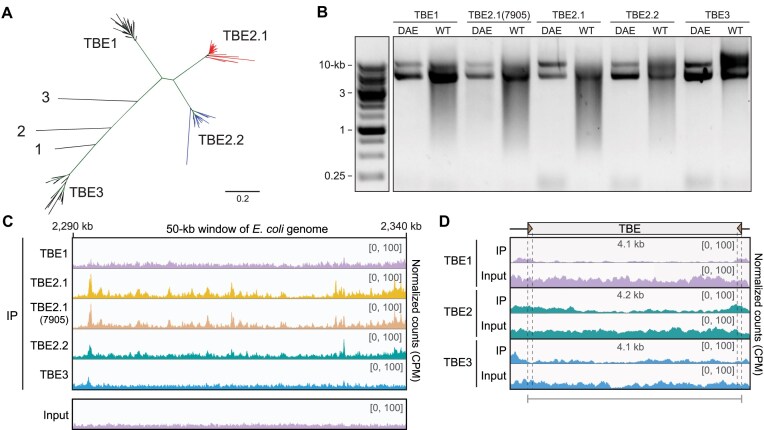
TBE transposase homologs are catalytically active and exhibit similar enrichment profiles across the *E. coli* genome. (**A**) Unrooted Bayesian phylogenetic tree of four families of TBE elements, from [6], built using the protein sequences of the 57-kDa protein. The scale indicates branch substitutions per site, and branches labeled 1–3 are the following TBE orthologs: (i) *Sterkiella histriomuscorum*; (ii) *Tetramena*sp.; (iii) *Laurentiella*sp. (**B**) DNA fragmentation assay for representative TBE transposase homologs from each of the four major families, upon heterologous expression in *E. coli*. Each sample was induced for 18 h before DNA was purified, and 500 ng DNA was separated by electrophoresis on a 1% agarose gel and visualized using SYBR Safe DNA Gel Stain. A catalytically inactivated mutant (DAE) was generated and cloned for a representative of each TBE family. For each transposase representative, only the WT transposase induces DNA fragmentation. (**C**) Read coverage across a representative 50-kb window of the *E. coli* genome from ChIP-seq using the catalytically inactivated representative from each TBE group. The *y*-axis represents CPM and the axis limit is set to 100. (**D**) Read coverage in CPM from ChIP-seq in strains harboring a genomically integrated TBE element corresponding to a representative from TBE1 (from ctg7180000067530), TBE2 (from ctg7180000069065), or TBE3 (from ctg7180000089059). The *y*-axis represents CPM with the axis limit set to 100, and dashed lines indicate the TIR boundaries.

Phylogenetic analysis of the 42-kDa transposase ORF encoded within diverse TBE transposons revealed that they cluster into four families—TBE1, TBE2.1, TBE2.2, and TBE3 (Fig. 4A) [6]—and allowed the generation of consensus protein sequences for each family that share >70% amino acid identity between families [6] and possess conserved catalytic active sites (Supplementary Fig. S5A–C). We wondered whether differences among transposase families might confer differences in transposase activity and therefore repeated the DNA fragmentation and ChIP-seq assays using the consensus sequences for each of the four TBE families (Fig. 4B–D). Each transposase representative induced a similar DNA fragmentation phenotype, although the phenotype was less dramatic for the TBE3 family sequence (Fig. 4B). We also prepared catalytically inactive variants of each transposase family sequence and confirmed that DNA fragmentation required the presence of an intact catalytic triad. ChIP-seq experiments using the consensus sequences revealed consistent profiles of DNA enrichment across the *E. coli* genome, although the profiles for the TBE1 and TBE3 representatives were less pronounced (Fig. 4C). The most noticeable difference when using consensus sequences arose when we performed ChIP-seq in *E. coli* strains encoding one particular copy of a genomically integrated TBE transposon from each family. In these genomic backgrounds, we did not detect strong enrichment within the transposon TIRs (Fig. 4D). This result could be attributed to differences in the DNA binding preference, or to differences between the transposon TIRs among families, which differ up to 47% [6] (Supplementary Fig. S5D).

### Transposase binding correlates with DNA cleavage

**Figure 5.**
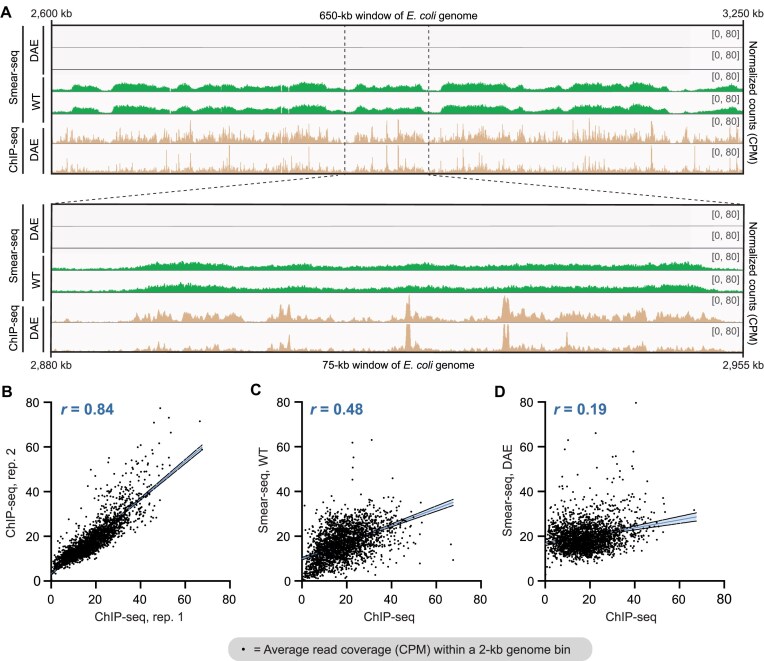
Transposase binding activity (ChIP-seq) correlates with cleavage activity (Smear-seq). (**A**) Read coverage profiles of the 18-h Smear-seq DAE and WT samples, compared to the 18-h ChIP-seq DAE (catalytically inactivated) signal, in 650-kb (top) or 80-kb (bottom) bins across the *E. coli* genome. The *y*-axis represents CPM with the axis limit set to 80. (B-D) Comparison between average read coverage (CPM) across 2-kb bins of the *E. coli* genome. There is strong correlation between ChIP-seq replicates (**B**), moderate correlation between ChIP-seq DAE and 18-h WT Smear-seq samples (**C**), and no correlation between the ChIP-seq DAE and the 18 h DAE Smear-seq signal (**D**). *r* values represent the Pearson correlation, and linear regressions are shown with 95% confidence bands of the best-fit line.

We wondered whether the DNA signatures from our ChIP-seq and Smear-seq approaches overlapped, which would indicate correspondence between DNA binding and cleavage activity. Visual inspection of the read coverage profiles from both datasets revealed an apparent consistency between the ChIP-seq and 18-h WT Smear-seq samples, despite the more diffuse mapping in the Smear-seq profiles compared to the ChIP-seq profiles, which could be due to downstream DNA degradation after transposase-mediated DNA cleavage (Fig. 5A and Supplementary Fig. S6A). To test whether this qualitative similarity was statistically significant, we averaged the genome-wide read coverage within 2-kb bins and compared the average coverage between samples (Fig. 5B–D and Supplementary Fig. S6B). This analysis revealed a moderate positive correlation (*r =*0.48) between the ChIP-seq signal and the Smear-seq signal after 18 h of inducing the catalytically active transposase (Fig. 5C). The moderate correlation disappeared when we compared the ChIP-seq signal to the Smear-seq signal after inducing the catalytically inactive transposase (Fig. 5D). We also observed a remarkable consistency between Smear-seq replicates at 18 h using the WT transposase (*r* = 0.99, Supplementary Fig. S6B), reflecting reproducible and thorough DNA degradation in the presence of catalytically active *Oxytricha* transposase expressed in *E. coli*, confirming efficient expression. The correlation patterns remained consistent across all bin sizes ranging from 500 bp to 10 kb (Supplementary Fig. S6C). Overall, the consistency between regions of DNA binding, as measured by ChIP-seq, and regions of DNA cleavage, as measured by Smear-seq, indicates that the DNA recognition and cleavage activity of the TBE transposase is sequence-specific, favoring degenerate motifs that are partially present within the TBE transposon ends.

### TBE transposase is expressed in the developing nucleus and preferentially associates with TBE elements in *Oxytricha*

Efforts to study the biochemical properties of TBE transposases *in vitro* have been hampered by protein insolubility (Orsolya Barabas, personal communication), so we decided to pursue alternative experiments to monitor its native localization and activity in *Oxytricha*. We selected a 14-amino-acid peptide corresponding to the predicted surface-exposed region between the two N-terminal DNA binding domains (Supplementary Fig. S7A), which is conserved in TBE2.1 and TBE2.2 (Supplementary Fig. S7B), and purified polyclonal antibodies after rabbit immunization. Western hybridization upon heterologous TBE transposase expression in *E. coli* revealed a clear band at the expected size (Supplementary Fig. S7C), supporting the specificity of the antibody.

**Figure 6.**
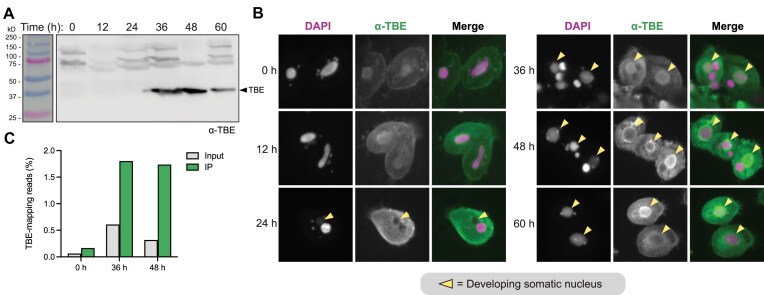
TBE transposase expression, localization, and transposon-DNA preference during *Oxytricha* development. (**A**) Western blot analysis using an anti-TBE transposase antibody against *Oxytricha* cell lysates collected at 12-h intervals after mixing of compatible mating types detects specific protein expression at the expected size of 42 kDa, starting at 36 h. (**B**) DAPI staining of DNA and anti-TBE transposase immunostaining of *Oxytricha* cells at 12-h intervals post-mixing. Arrowheads indicate developing somatic macronuclei. Note that until 48 h, DAPI stains the parental macronuclei more strongly than the new macronuclei, due to the copy number difference. (**C**) Percent of micronuclear genome-mapping ChIP-seq reads that map to TBE2.1 (7905) in the input and IP samples, at 0 h and two time points after mixing.

Next, we performed a mating time course of *Oxytricha* cells and collected lysates at 12-h intervals for anti-TBE transposase western analysis to monitor transposase expression. A strong 42-kDa band was present at 36 and 48 h, corresponding to the expected peak of genome rearrangement (Fig. 6A) [4, 14]. To investigate TBE transposase localization, we performed immunofluorescence at the same 12-h intervals post-mixing and observed a strong signal in developing somatic nuclei (Fig. 6B). These data reveal that TBE transposases are not only expressed during development, but that they specifically localize to *Oxytricha*’s developing somatic macronucleus.

Finally, we performed ChIP-seq experiments in *Oxytricha* cells at 0, 36, and 48 h post-mixing. When we mapped sequence reads back to the TBE family transposons and compared the proportion of reads mapping to TBEs versus to the remainder of the micronuclear genome, we observed an ∼4-fold enrichment compared to input, and a striking >8-fold enrichment of TBEs at developmental time points compared to the 0 h time point (Fig. 6C), revealing that TBE transposases preferentially target TBE transposon elements natively in *Oxytricha*. We did not observe peaks of enrichment within the transposon end sequences themselves (Supplementary Fig. S7D), which may be due to read-mapping artifacts; because we are mapping ChIP-seq reads to a single TBE2.1 (7905) element, the sequence variability flanking the high copy number TBEs heterogeneously distributed throughout the genome may cause coverage to drop out near the boundary of the element. Taken together, these data reveal that *Oxytricha* TBE transposases are expressed in their developing somatic nucleus, and preferentially associate with TBE-specific DNA transposons.

## Discussion

By leveraging a heterologous expression strategy in *E. coli*, our study provides the first insights into TBE transposase activity, revealing the enzyme’s DNA sequence preferences for binding and cleavage. Our heterologous expression approach addresses a critical gap in understanding the DNA substrate preferences of this enzyme that participates in genome rearrangement. Functional studies of the TBE transposase in *Oxytricha* have been limited by tools to manipulate a high-copy element in the germline genome. Our present approach overcomes these limitations and provides insight into the mechanism of DNA targeting and cleavage. The approach may be leveraged to study the activity of other transposases, which can be notoriously difficult to purify due to their basic properties and aggregation propensities [38], limiting the ability to study their biochemical properties and activities *in vitro*.

After observing an unexpected DNA fragmentation pattern when we expressed the TBE transposase in *E. coli*, we developed a deep sequencing approach (“Smear-seq”) to investigate the enzyme’s genomic DNA fragmentation activity. By leveraging an orthogonal approach to directly profile DNA binding sites of the catalytically inactive transposase using ChIP-seq and *de novo* motif predictions, we discovered that the transposase preferentially binds short sequence variations of the motif 5′-ANTTTGA, which is partially present within the TBE transposon ends. Moreover, ChIP-seq revealed enrichment of the transposase within the native TBE end sequences (TIRs) of the TBE2.1 (7905) variant. We further applied our scalable approach to profile DNA binding specificities for all major families of TBE transposases, revealing their ability to induce DNA fragmentation and to display consistent enrichment profiles across the genome. Finally, we found a moderately positive correlation between the ChIP-seq and Smear-seq signals, indicating that the TBE transposase not only binds DNA at specific sites but can also induce DNA fragmentation at these loci.

One limitation of the heterologous workflow is that it does not reconstitute native protein interactions between the TBE transposase and other *Oxytricha* elements that are absent from the *E. coli* expression system. We also acknowledge that our experiments primarily examined a single TBE2.1 transposase variant and consensus sequences from the four transposon families, which cannot represent the activity of the >34 000 specific TBE elements encoded in the MIC genome. Importantly, the majority of germline-encoded transposase genes do not contain an intact catalytic triad [6], indicating that they have lost DNA cleavage activity and could be pseudogenes. However, non-catalytic paralogs could also serve other functions as binding partners or interactors, as in another model ciliate, *Paramecium tetraurelia* [39].

An additional limitation is that ChIP-seq performed in *Oxytricha* yields broadly distributed signal across the genome, which makes it difficult to resolve enrichment at individual loci such as the *TEBPβ* locus. In contrast, ChIP-seq in *E. coli* offers higher resolution and reduced background, facilitating clearer detection of locus-specific enrichment patterns. While our bacterial expression system enables detailed characterization of binding profiles, future work will be needed to optimize native ChIP-seq approaches in *Oxytricha* to more precisely map transposase recruitment to specific genomic sites.

Transposases involved in genome rearrangement have been extensively profiled in other model ciliates, which also undergo large-scale genome rearrangements, albeit with reduced complexity and at a smaller scale than *Oxytricha*. *Paramecium* genome rearrangement eliminates >45 000 IESs and requires a domesticated PiggyMac (PGM) transposase [40, 41]. PGM is homologous to the *piggyBac* transposase, originally isolated from a cabbage looper moth, and it inserts precisely and exclusively into TTAA target sites [42]. In *Paramecium*, PGM is expressed in the developing macronucleus shortly after mating, and knockdown of PGM reduces cell viability and inhibits IES excision [41]. Not all copies of PiggyMac are catalytically intact; five groups of catalytically inactivated Pgm-like proteins (Pgmls) interact with PiggyMac, and are required for both nuclear localization and DNA elimination activity [39]. Intriguingly, the DNA-targeting activity of the catalytically intact PGM is influenced by chromatin state, and the nucleosome remodeling protein ISWI1 participates in IES nucleosome depletion, perhaps by increasing chromatin accessibility for DNA cleavage by PGM [43]. Although our work in *Oxytricha* indicates that the TBE transposase is sequence-specific, we have yet to determine the extent to which chromatin state modulates transposase recruitment or cleavage activity.

The genome of another well-studied ciliate, *Tetrahymena*, also encodes domesticated *piggyBac*-like transposases that are implicated in DNA rearrangement. Three of these transposases, Tpb1, Tpb2, and Tpb6, are also expressed shortly after mating and their knockdown impairs viability and inhibits DNA elimination [44, 45]. The targeting mechanism of Tpb2 is based on recruitment to heterochromatin structures, as opposed to specific sequences [44], while Tpb1/6 are thought to require sequence recognition at the boundaries of eliminated sequences [45]. Future work is needed to determine whether DNA recruitment of the TBE transposase is dependent on heterochromatin structures in its native environment; however, the short length of many *Oxytricha* IESs presents a challenge to chromatin recognition [4].

The relaxed sequence specificity of the TBE transposases differs markedly from the strict sequence-specific activity of most transposases that exclusively target their transposon end sequences to minimize off-target cleavage activity that could compromise host fitness. Although our work demonstrates that the TBE transposases do recognize their end sequences, they also exhibit remarkable off-target activity, with the ability to reproducibly bind and cleave distinct regions across the *E. coli* genome. Future work in *Oxytricha* is needed to confirm whether this relaxed specificity is indeed critical to facilitate the extensive cleavage events that occur during development of the somatic macronucleus.

An alternative model suggested by our data is that the TBE transposase primarily functions as a genome-fragmenting enzyme with relatively relaxed sequence specificity, while the precise definition of MDS–IES boundaries occurs later during the reassembly phase. Our observation of widespread, reproducible DNA fragmentation, together with prior evidence of imprecisely joined excised IES circles [13], supports the plausibility of this model. Future studies will be needed to determine whether transposase-mediated cleavage and sequence-directed reassembly are mechanistically separable steps during *Oxytricha* genome rearrangement.

Together, this work reveals DNA binding and cleavage activity of the TBE transposases involved in *Oxytricha* genome rearrangement, and describes a facile heterologous approach to profile the activity of enzymes implicated in DNA rearrangements.

## Supplementary Material

gkaf577_Supplemental_Files

## Data Availability

High-throughput sequencing data and custom scripts used for analyses of high-throughput sequencing data are available on Zenodo (DOI:10.5281/zenodo.15040686). Sequencing data are also available at the National Center for Biotechnology Information (NCBI) Sequence Read Archive (BioProject accession: PRJNA1237807). Datasets generated and analyzed in the current study are available from the corresponding authors on request.

## References

[1] Wang J, Davis RE Programmed DNA elimination in multicellular organisms. Curr Opin Genet Dev. 2014; 27:26–34. 10.1016/j.gde.2014.03.012. 24886889 PMC4125452

[2] Smith JJ, Timoshevskiy VA, Saraceno C Programmed DNA elimination in vertebrates. Annu Rev Anim Biosci. 2020; 9:173–201. 32986476 10.1146/annurev-animal-061220-023220 PMC8715500

[3] Prescott DM The DNA of ciliated protozoa. Microbiol Rev. 1994; 58:233–67. 10.1128/mr.58.2.233-267.1994. 8078435 PMC372963

[4] Chen X, Bracht JR, Goldman AD et al. The architecture of a scrambled genome reveals massive levels of genomic rearrangement during development. Cell. 2014; 158:1187–98. 10.1016/j.cell.2014.07.034. 25171416 PMC4199391

[5] Swart EC, Bracht JR, Magrini V et al. The *Oxytricha trifallax* macronuclear genome: a complex eukaryotic genome with 16,000 tiny chromosomes. PLoS Biol. 2013; 11:e1001473 10.1371/journal.pbio.1001473. 23382650 PMC3558436

[6] Chen X, Landweber LF Phylogenomic analysis reveals genome-wide purifying selection on TBE transposons in the ciliate *Oxytricha*. Mobile DNA. 2016; 7:2 10.1186/s13100-016-0057-9. 26811739 PMC4724952

[7] Lindblad KA, Pathmanathan JS, Moreira S et al. Capture of complete ciliate chromosomes in single sequencing reads reveals widespread chromosome isoforms. BMC Genomics. 2019; 20:1037 10.1186/s12864-019-6189-9. 31888453 PMC6937825

[8] Fang W, Wang X, Bracht JR et al. Piwi-interacting RNAs protect DNA against loss during *Oxytricha* genome rearrangement. Cell. 2012; 151:1243–55. 10.1016/j.cell.2012.10.045. 23217708 PMC3678556

[9] Nowacki M, Vijayan V, Zhou Y et al. RNA-mediated epigenetic programming of a genome-rearrangement pathway. Nature. 2008; 451:153–8. 10.1038/nature06452. 18046331 PMC2647009

[10] Nowacki M, Higgins BP, Maquilan GM et al. A functional role for transposases in a large eukaryotic genome. Science. 2009; 324:935–8. 10.1126/science.1170023. 19372392 PMC3491810

[11] Doak TG, Doerder FP, Jahn CL et al. A proposed superfamily of transposase genes: transposon-like elements in ciliated protozoa and a common “D35E” motif. Proc Natl Acad Sci USA. 1994; 91:942–6. 10.1073/pnas.91.3.942. 8302872 PMC521429

[12] Hickman AB, Dyda F DNA transposition at work. Chem Rev. 2016; 116:12758–84. 10.1021/acs.chemrev.6b00003. 27187082 PMC6380494

[13] Yerlici VT, Lu MW, Hoge CR et al. Programmed genome rearrangements in *Oxytricha* produce transcriptionally active extrachromosomal circular DNA. Nucleic Acids Res. 2019; 47:9741–60. 10.1093/nar/gkz725. 31504770 PMC6765146

[14] Williams K, Doak TG, Herrick G Developmental precise excision of *Oxytricha trifallax* telomere-bearing elements and formation of circles closed by a copy of the flanking target duplication. EMBO J. 1993; 12:4593–601. 8223469 10.1002/j.1460-2075.1993.tb06148.x PMC413894

[15] Klobutcher LA, Herrick G Developmental genome reorganization in ciliated protozoa: the transposon link. Prog Nucleic Acid Res Mol Biol. 1997; 56:1–62. 9187050 10.1016/s0079-6603(08)61001-6

[16] Clay DM, Kim H, Landweber LF Transformation with artificial chromosomes in *Oxytricha trifallax* and their applications. G3: genes, genomes, genet. 2019; 9:3119–27. 10.1534/g3.119.400298. PMC6778790 31506318

[17] Kataoka K, Schoeberl UE, Mochizuki K Modules for C-terminal epitope tagging of *Tetrahymena* genes. J Microbiol Methods. 2010; 82:342–6. 10.1016/j.mimet.2010.07.009. 20624430 PMC2935961

[18] Jumper J, Evans R, Pritzel A et al. Highly accurate protein structure prediction with AlphaFold. Nature. 2021; 596:583–9. 10.1038/s41586-021-03819-2. 34265844 PMC8371605

[19] Evans R, O’Neill M, Pritzel A et al. Protein complex prediction with AlphaFold-Multimer. bioRxiv10 March 2022, preprint: not peer reviewed 10.1101/2021.10.04.463034.

[20] Baek M, McHugh R, Anishchenko I et al. Accurate prediction of protein–nucleic acid complexes using RoseTTAFoldNA. Nat Methods. 2024; 21:117–21. 10.1038/s41592-023-02086-5. 37996753 PMC10776382

[21] Madeira F, Pearce M, Tivey ARN et al. Search and sequence analysis tools services from EMBL-EBI in 2022. Nucleic Acids Res. 2022; 50:W276–9. 10.1093/nar/gkac240. 35412617 PMC9252731

[22] Waterhouse AM, Procter JB, Martin DM et al. Jalview version 2—a multiple sequence alignment editor and analysis workbench. Bioinformatics. 2009; 25:1189–91. 10.1093/bioinformatics/btp033. 19151095 PMC2672624

[23] Chen S, Zhou Y, Chen Y et al. fastp: an ultra-fast all-in-one FASTQ preprocessor. Bioinformatics. 2018; 34:i884–90. 10.1093/bioinformatics/bty560. 30423086 PMC6129281

[24] Langmead B, Salzberg SL Fast gapped-read alignment with Bowtie 2. Nat Methods. 2012; 9:357–9. 10.1038/nmeth.1923. 22388286 PMC3322381

[25] Danecek P, Bonfield JK, Liddle J et al. Twelve years of SAMtools and BCFtools. GigaScience. 2021; 10:giab008 10.1093/gigascience/giab008. 33590861 PMC7931819

[26] Ramírez F, Ryan DP, Grüning B et al. deepTools2: a next generation web server for deep-sequencing data analysis. Nucleic Acids Res. 2016; 44:W160–5. 10.1093/nar/gkw257. 27079975 PMC4987876

[27] Walker MWG, Klompe SE, Zhang DJ et al. Novel molecular requirements for CRISPR RNA-guided transposition. Nucleic Acids Res. 2023; 51:4519–35. 10.1093/nar/gkad270. 37078593 PMC10201428

[28] Bonocora RP, Wade JT ChIP-seq for genome-scale analysis of bacterial DNA-binding proteins. Methods Mol Biol. 2015; 1276:4519–35. 10.1007/978-1-4939-2392-2_20 25665574

[29] Park PJ ChIP-seq: advantages and challenges of a maturing technology. Nat Rev Genet. 2009; 10:669–80. 10.1038/nrg2641. 19736561 PMC3191340

[30] Hoffmann FT, Kim M, Beh LY et al. Selective TnsC recruitment enhances the fidelity of RNA-guided transposition. Nature. 2022; 609:384–93. 10.1038/s41586-022-05059-4. 36002573 PMC10583602

[31] Zhang Y, Liu T, Meyer CA et al. Model-based analysis of ChIP-Seq (MACS). Genome Biol. 2008; 9:R137 10.1186/gb-2008-9-9-r137. 18798982 PMC2592715

[32] Heinz S, Benner C, Spann N et al. Simple combinations of lineage-determining transcription factors prime *cis*-regulatory elements required for macrophage and B cell identities. Mol Cell. 2010; 38:576–89. 10.1016/j.molcel.2010.05.004. 20513432 PMC2898526

[33] Bryan G, Garza D, Hartl D Insertion and excision of the transposable element *mariner* in *Drosophila*. Genetic. 1990; 125:103–14. 10.1093/genetics/125.1.103 PMC1203992 2160399

[34] Bryan GJ, Jacobson JW, Hartl DL Heritable somatic excision of a *Drosophila* transposon. Science. 1987; 235:1636–8. 10.1126/science.3029874. 3029874

[35] Richardson JM, Colloms SD, Finnegan DJ et al. Molecular architecture of the *Mos1* paired-end complex: the structural basis of DNA transposition in a eukaryote. Cell. 2009; 138:1096–108. 10.1016/j.cell.2009.07.012. 19766564 PMC3977044

[36] Trubitsyna M, Grey H, Houston DR et al. Structural basis for the inverted repeat preferences of *mariner* transposases. J Biol Chem. 2015; 290:13531–40. 10.1074/jbc.M115.636704. 25869132 PMC4505599

[37] Dupeyron M, Baril T, Bass C et al. Phylogenetic analysis of the Tc1/*mariner* superfamily reveals the unexplored diversity of pogo-like elements. Mobile DNA. 2020; 11:21 10.1186/s13100-020-00212-0. 32612713 PMC7325037

[38] Jaillet J, Dussaussois-Montagne A, Renault S et al. Expression and purification of the eukaryotic MBP-MOS1 transposase from *sf21* insect cells. Bio-Protocol. 2014; 4:e1262 10.21769/BioProtoc.1262.

[39] Bischerour J, Bhullar S, Wilkes CD et al. Six domesticated PiggyBac transposases together carry out programmed DNA elimination in *Paramecium*. eLife. 2018; 7:e37927 10.7554/eLife.37927. 30223944 PMC6143343

[40] Arnaiz O, Mathy N, Baudry C et al. The *Paramecium* germline genome provides a niche for intragenic parasitic DNA: evolutionary dynamics of internal eliminated sequences. PLoS Genet. 2012; 8:e1002984 10.1371/journal.pgen.1002984. 23071448 PMC3464196

[41] Baudry C, Malinsky S, Restituito M et al. PiggyMac, a domesticated *piggyBac* transposase involved in programmed genome rearrangements in the ciliate *Paramecium tetraurelia*. Genes Dev. 2009; 23:2478–83. 10.1101/gad.547309. 19884254 PMC2779751

[42] Fraser MJ, Smith GE, Summers MD Acquisition of host cell DNA sequences by baculoviruses: relationship between host DNA insertions and FP mutants of *Autographa californica* and *Galleria mellonella* nuclear polyhedrosis viruses. J Virol. 1983; 47:287–300. 16789244 10.1128/jvi.47.2.287-300.1983 PMC255260

[43] Singh A, Maurer-Alcalá XX, Solberg T et al. Chromatin remodeling is required for sRNA-guided DNA elimination in *Paramecium*. EMBO J. 2022; 41:EMBJ2022111839 10.15252/embj.2022111839. PMC9670198 36221862

[44] Cheng C-Y, Vogt A, Mochizuki K et al. A domesticated *piggyBac* transposase plays key roles in heterochromatin dynamics and DNA cleavage during programmed DNA deletion in *Tetrahymena thermophila*. Mol Biol Cell. 2010; 21:1753–62. 10.1091/mbc.e09-12-1079. 20357003 PMC2869380

[45] Cheng C-Y, Young JM, Lin C-YG et al. The *piggyBac* transposon-derived genes TPB1 and TPB6 mediate essential transposon-like excision during the developmental rearrangement of key genes in *Tetrahymena thermophila*. Genes Dev. 2016; 30:2724–36. 10.1101/gad.290460.116. 28087716 PMC5238731

