# Supplementary material for: Relaxed DNA substrate specificity of transposases involved in programmed genome rearrangement
Source: Nucleic Acids Res. 2025 Jul 10;53(13):gkaf577. doi: 10.1093/nar/gkaf577 (PMC12242761; doi:10.1093/nar/gkaf577)
Supplement: gkaf577_Supplemental_Files [file gkaf577_supplemental_files.zip › Walker_etal_2025-NAR_Supplementary_Figures.pdf]

SUPPLEMENTARY FIGURES

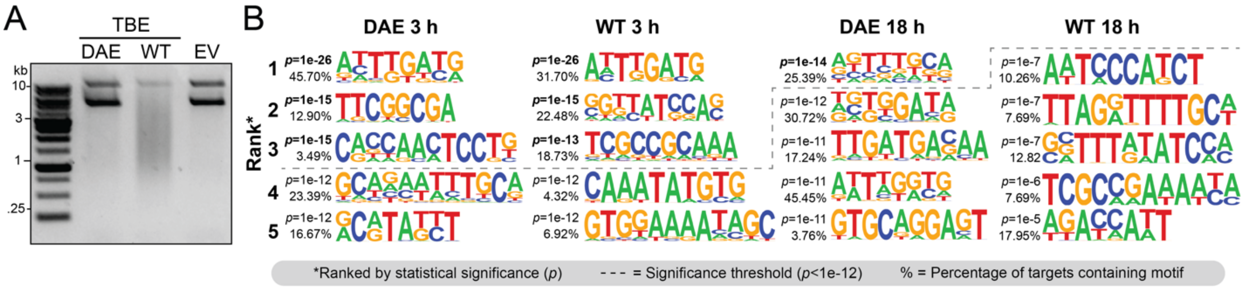

Figure S1 | TBE transposase fragmentation controls and *de novo* motif analysis

(**A**) DNA fragmentation analysis upon TBE transposase induction in *E. coli*, using either a catalytically-inactivated transposase (DAE), the WT transposase, or an empty vector (EV). 500 ng DNA was separated by electrophoresis on a 1% agarose gel and visualized using SYBR Safe. (**B**) Sequence logos identified from *de novo* motif prediction (Homer; (1)) within peaks of ChIP-seq read enrichment for the catalytically-inactivated (DAE) and WT transposase. Sequence logos are ranked by statistical significance, and the top five most significant motifs from each sample are shown. The dashed line indicates the cutoff for statistical significance (*p*-value<1e-12). The percentage of ChIP-seq peaks containing each motif is listed to the left of each motif (%).

Figure S2

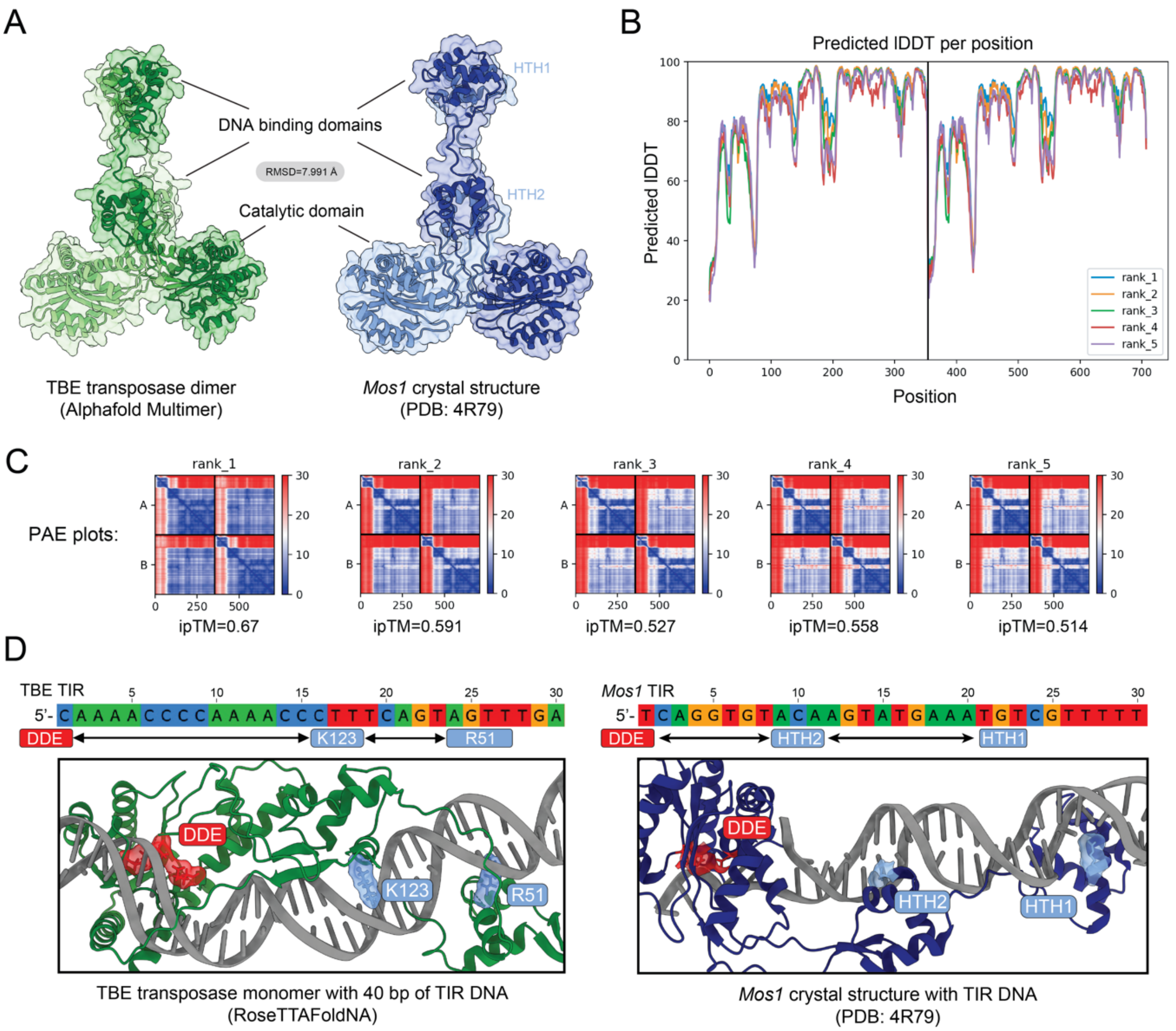

Figure S2 | Structural prediction of the TBE transposase places putative DNA binding domains near preferred motifs within transposon ends. (**A**) AlphaFold-Multimer (2) prediction of the TBE2.1(7905) transposase dimer (left). Annotated DNA binding domains are based on structural homology to the *Mos1* crystal structure (right) (3) . DNA-binding helix-turn-helix (HTH) and catalytic domains are indicated. (**B-C**) Model quality metrics for the TBE transposase dimer prediction shown in (A). (**B**) Per-residue predicted Local Distance Difference Test (pLDDT) confidence scores for the “rank_1” model, which was selected for analysis and shown in (A). (**C**) Predicted Aligned Error (PAE) heatmaps showing the estimated positional uncertainty between pairs of residues across the dimer structural predictions. (**D**) Left: RoseTTAFoldNA (4) prediction of a TBE transposase monomer interacting with TBE TIR DNA. The TIR DNA schematic (top)

illustrates the predicted interaction sites of the DNA binding domain residues (K123 and R51) based on the RoseTTAFoldNA model. Right: Crystal structure of the *Mos1* transposase bound to its cognate transposon end DNA (3), illustrating the regions contacted by the N-terminal helix-turn-helix DNA binding domains (HTH1, HTH2).

Figure S3

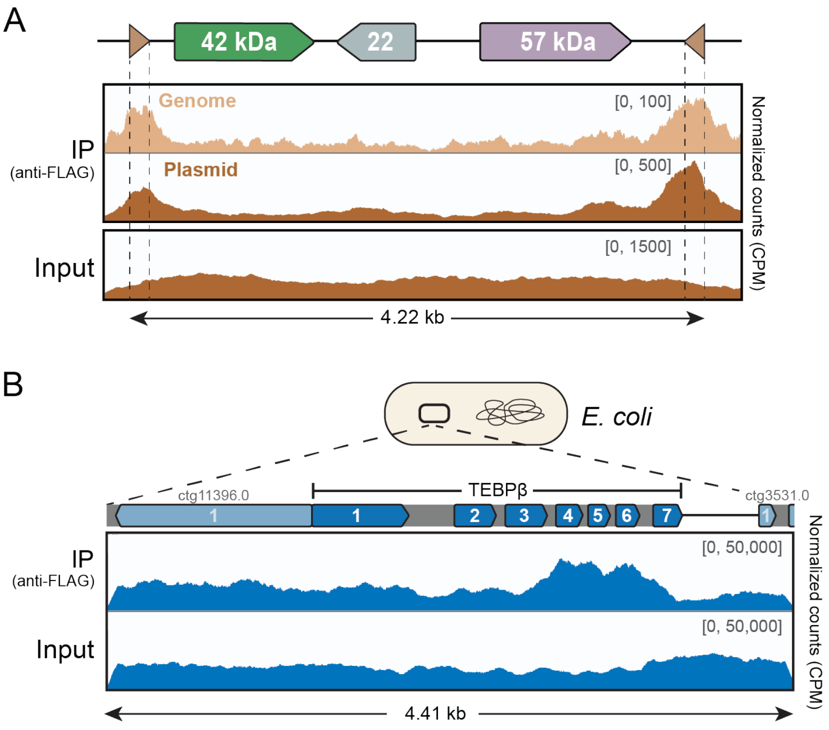

Figure S3 | The TBE transposase does not exhibit a consistent pattern of enrichment across *Oxytricha* MIC genomic DNA in *E. coli*. (**A**) Read coverage from ChIP-seq of the TBE transposase using either a genomically-integrated ("genome") or a plasmid-encoded complete TBE transposon ("plasmid") in *E. coli*. The y-axis represents CPM and is set to the following maximum for each sample: 100 CPM for the genome IP track; 500 CPM for the plasmid IP track; and 1500 CPM for the plasmid input track. (**B**) ChIP-seq read coverage from the FZZ-tagged TBE transposase expressed in *E. coli* cells containing a cloned *Oxytricha* MIC genomic region on a plasmid containing the TEBPβ MIC locus and flanking regions. The y-axis represents CPM and is set to 50,000 for both the IP and input samples. Dark blue boxes within the TEBPβ MIC locus depict its 7 MDSs, gray boxes represent IESs, and pale blue boxes are MDSs for flanking loci; figure approximately to scale.

Figure S4

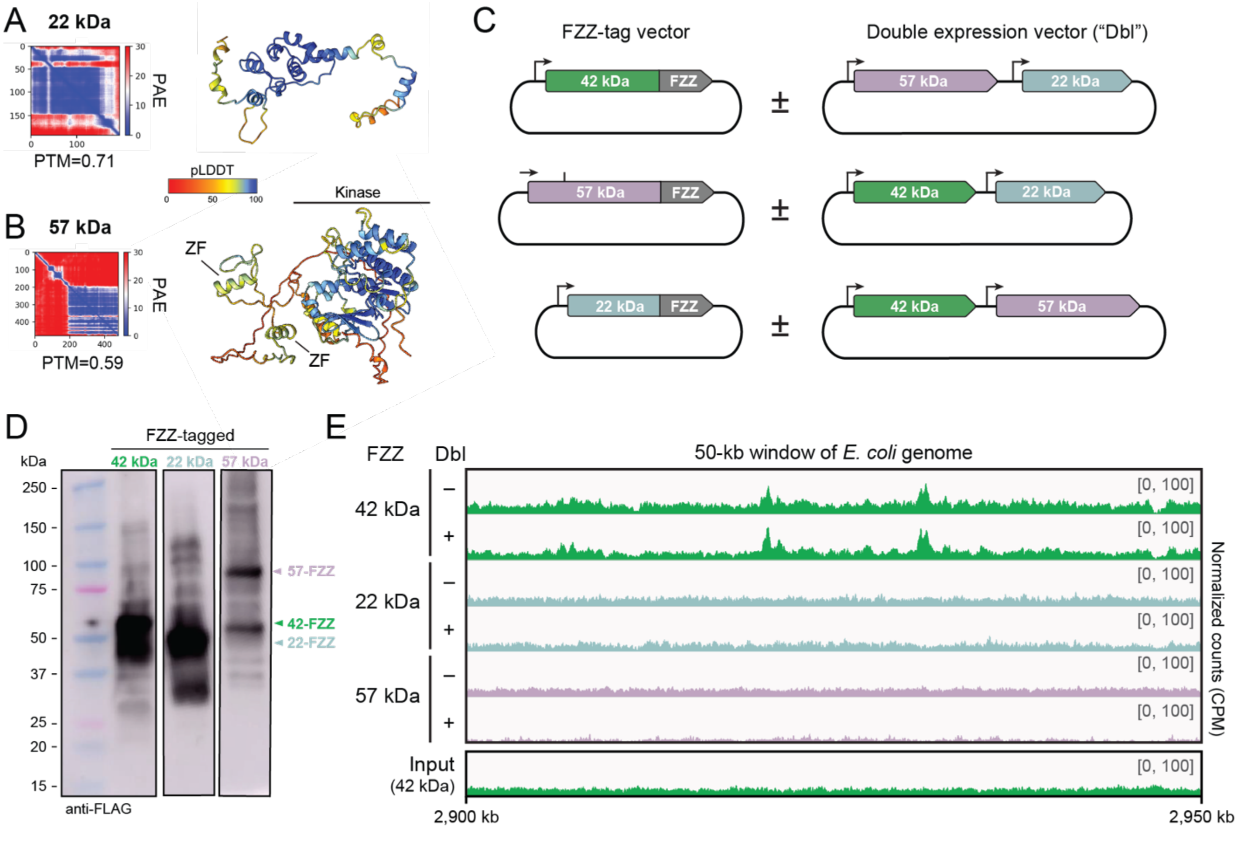

Figure S4 | Investigating the accessory proteins encoded within TBE transposons. (**A**) Left: AlphaFold prediction of the 22-kDa structure, colored by pLDDT (predicted Local Distance Difference Test) confidence scores. No high-confidence domain predictions were identified by HHpred (5). Right: Predicted Aligned Error (PAE) plot for the 22-kDa ORF showing estimated positional uncertainty between residue pairs. (**B**) Left: AlphaFold prediction of the 57-kDa structure colored by pLDDT scores. Predicted zinc-finger (ZF) and kinase domains, identified using HHpred, are annotated. (**C**) Schematic of expression vector design for ChIP-seq experiments in D. In each experiment (horizontal rows in D), strains contained pairwise combinations of FZZ-tagged single-protein vectors in the presence and absence of untagged double protein expression vectors ('Dbl'). (**D**) Western blot with an anti-FLAG antibody used against each of the three FZZ-tagged expression vectors in *E. coli*. Expected sizes for the FZZ-fusion proteins are 77 kDa (57-kDa ORF), 60 kDa (42-kDa ORF), and 42 kDa (22-kDa ORF). (**E**) Read coverage across a representative 60-kb window of the *E. coli* genome for the FZZ-tagged expression vectors, in the presence (+) and absence (-) of the corresponding untagged double-

expression vectors (‘Dbl’). The y-axis represents counts per million (CPM), and the axis limit is set to 100.

Figure S5

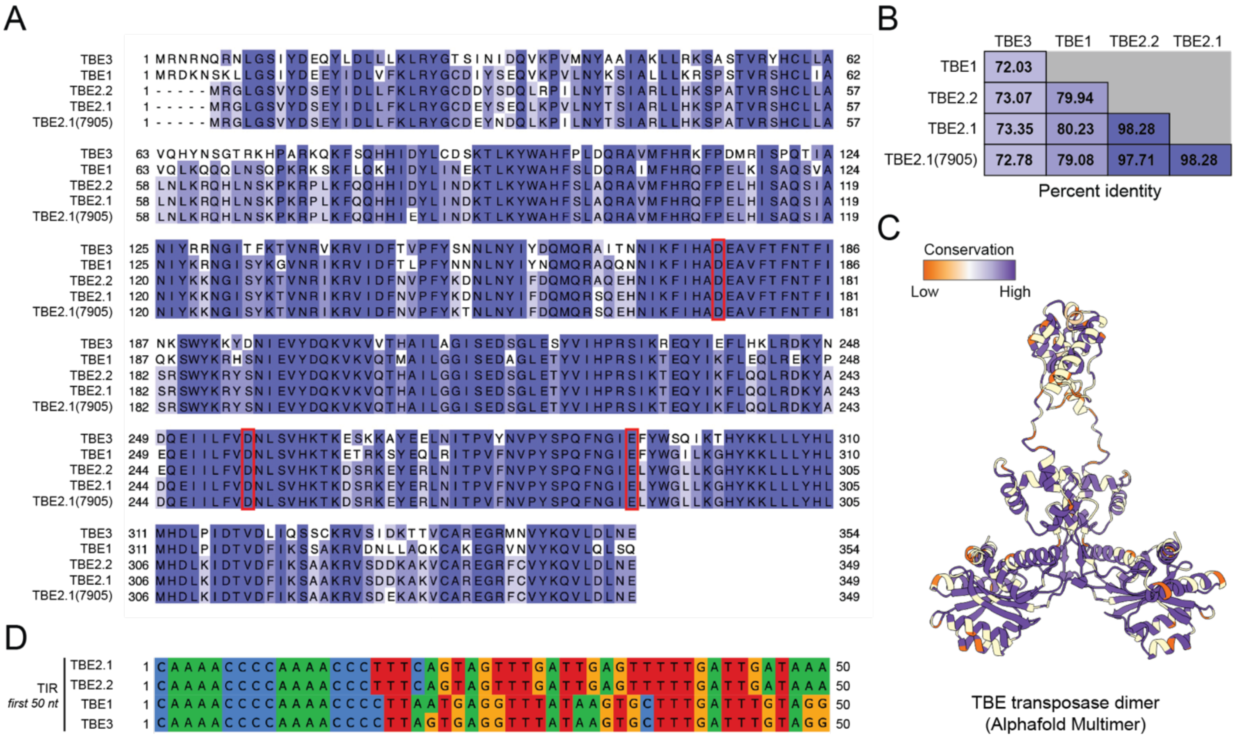

Figure S5 | Alignment and amino acid similarity across TBE transposase families. (**A**) Multiple sequence alignment of the four TBE family transposase consensus sequences (Chen *et al*. 2016), alongside representative TBE2.1 family member (7905). Red boxes indicate residues belonging to the DDE catalytic triad. (**B**) Sequence identity matrix of the percent amino acid identity for the four TBE family consensus sequences and TBE2.1(7905). (**C**) TBE transposase dimer structural prediction colored by sequence conservation across the TBE families. (**D**) Alignment of the first 50 nt of the TIRs within each family, adapted from X. Chen & Landweber, 2016.

Figure S6

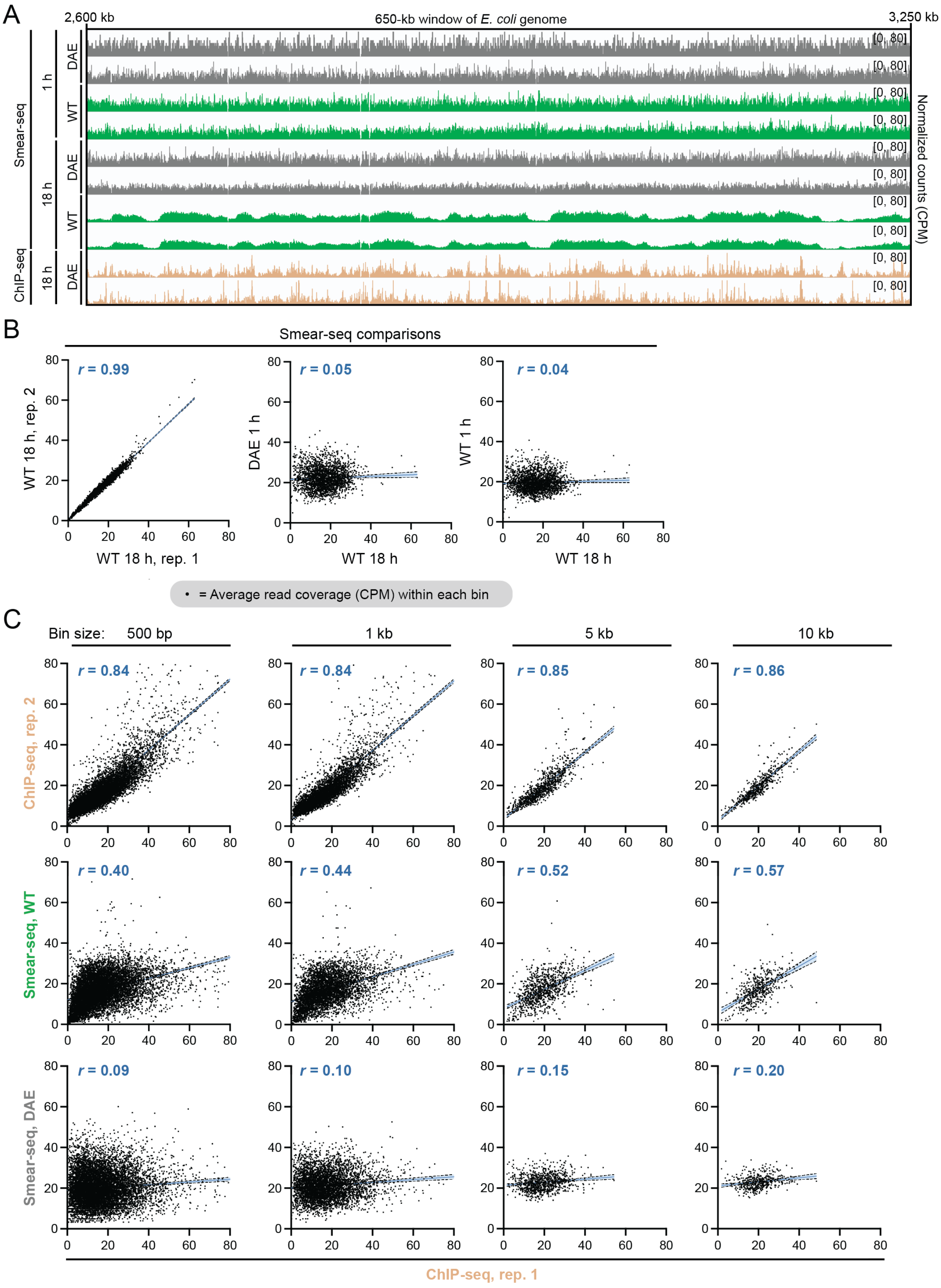
A
2,600 kb
650-kb window of E. coli genome
3,250 kb
Smear-seq
1 h
DAE
WT
18 h
DAE
WT
ChIP-seq
18 h
DAE
[0, 80]
Normalized counts (CPM)
B
Smear-seq comparisons
r = 0.99
WT 18 h, rep. 2
WT 18 h, rep. 1
r = 0.05
DAE 1 h
WT 18 h
r = 0.04
WT 1 h
WT 18 h
• = Average read coverage (CPM) within each bin
C
Bin size: 500 bp
1 kb
5 kb
10 kb
r = 0.84
r = 0.84
r = 0.85
r = 0.86
ChIP-seq, rep. 2
r = 0.40
r = 0.44
r = 0.52
r = 0.57
Smear-seq, WT
r = 0.09
r = 0.10
r = 0.15
r = 0.20
Smear-seq, DAE
ChIP-seq, rep. 1

Figure S6 (previous page) **| Comparison between ChIP-seq and Smear-seq datasets.** (**A**) Read coverage profiles of all Smear-seq samples and the 18 h ChIP-seq DAE sample, in a 650-kb window of the *E. coli* genome. The y-axis represents CPM with the axis limit set to 80. (**B**) Comparisons between average read coverage in CPM across 2-kb bins of the genome. The Smear-seq signal at 18 h is strongly correlated across independent biological replicates. (**C**) Coverage comparisons when bin sizes are varied between 500 bp and 10 kb. *r* values represent the Pearson correlation, and linear regressions are shown with 95% confidence bands of the best-fit line.

Figure S7

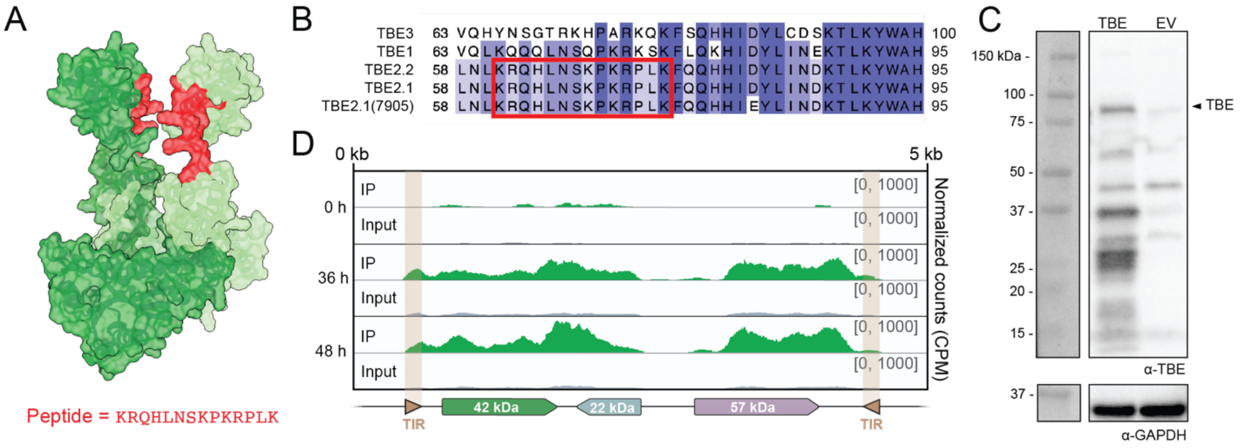

Figure S7 | Generating and validating an anti-TBE transposase antibody. (**A**) Structural prediction of the TBE2.1 transposase dimer by AlphaFold-Multimer (2); red coloring indicates the predicted surface-exposed region that was selected for peptide synthesis and antibody generation. (**B**) Partial multiple sequence alignment of the four TBE-family transposase consensus sequences (from **Figure S5A**). Red box indicates the 14 residues selected for peptide synthesis. (**C**) Western hybridization to the anti-TBE transposase antibody (top) and anti-GAPDH antibody loading control (bottom). The two samples are *E. coli* BL12(DE3) cells expressing either the MBP-tagged 42-kDa plasmid (TBE) or an empty vector control (EV). The expected size of the MBP-tagged 42-kDa ORF is 88.6 kDa. (**D**) Read coverage mapped across the representative TBE2.1(7905) element demonstrates transposon localization, concentrated at genic regions, at 36 and 48 h. The y-axis represents counts per million (CPM), and the axis limit is set to 1000.

REFERENCES

1. Heinz S, Benner C, Spann N *et al*. Simple combinations of lineage-determining transcription factors prime *cis*-regulatory elements required for macrophage and B cell identities. *Mol Cell* 2010;**38**:576–89. https://doi.org/10.1016/j.molcel.2010.05.004

2. Evans R, O'Neill M, Pritzel A *et al*. Protein complex prediction with AlphaFold-Multimer. bioRxiv, https://doi.org/10.1101/2021.10.04.463034, 10 March 2022, preprint: not peer reviewed.

3. Trubitsyna M, Grey H, Houston DR *et al*. Structural basis for the inverted repeat preferences of *mariner* transposases. *J Biol Chem* 2015;**290**:13531–40. https://doi.org/10.1074/jbc.M115.636704

4. Baek M, McHugh R, Anishchenko I *et al*. Accurate prediction of protein–nucleic acid complexes using RoseTTAFoldNA. *Nat Methods* 2024;**21**:117–21. https://doi.org/10.1038/s41592-023-02086-5

5. Söding J, Biegert A, Lupas AN. The HHpred interactive server for protein homology detection and structure prediction. *Nucleic Acids Res* 2025;**33**:W244–W248. https://doi.org/10.1093/nar/gki408
